# Effect of ATG initiation codon context motifs on the efficiency of translation of mRNA derived from exogenous genes in the transgenic silkworm, *Bombyx mori*

**DOI:** 10.1186/2193-1801-3-136

**Published:** 2014-03-10

**Authors:** Ken-ichiro Tatematsu, Keiro Uchino, Hideki Sezutsu, Toshiki Tamura

**Affiliations:** Transgenic Silkworm Research Unit, National Institute of Agrobiological Sciences, Tsukuba, Ibaraki 305-8634 Japan

**Keywords:** Kozak sequence, Translation, Silkworm, Transgenic, Recombinant protein, Bioreactor

## Abstract

The context sequence motif surrounding the ATG initiation codon influences mRNA translation efficiency and affects protein production; however, the optimal sequence differs among species. To determine the optimal sequence for production of recombinant proteins in a transgenic silkworm, we compared 14-nucleotide context motifs around the ATG (ATG-context) in 50 silkworm genes and found the following consensus: (A/T)AN(A/T)ATCAAAatgN. We were also able to define the least-common motif: CCN(C/G)CGN(C/T/G)(G/C/T)(T/G)atgC, which served as a negative control. To examine the regulatory role of these motifs in protein expression, we constructed reporter plasmids containing different ATG-context motifs together with either the luciferase gene or an enhanced green fluorescent protein (EGFP) gene. These constructs were then used for comparison of luciferase reporter activity and EGFP production in BmN4 cells *in vitro* as well as in transgenic silkworms *in vivo*. We detected 10-fold higher luciferase activity in BmN4 cells transfected with the consensus ATG-context motif construct, compared to the negative control plasmid. ELISA measurements of EGFP translation products with the corresponding constructs in BmN4 cells showed consistently similar results. Interestingly, the translation efficiency of the novel consensus ATG-context motif did not show the highest activity in the transgenic silkworms *in vivo*, except for the fat body. The highest efficiency in the middle and posterior silk glands was produced by the sericin 1 context. Our results show that the ATG-context motifs differ among silkworm tissues. This result is important for the further improvement of the transgenic silkworm system for the production of recombinant proteins.

## Background

There is an increased demand to develop an efficient bioreactor for the production of recombinant proteins for pharmaceutical and/or diagnostic uses. Transgenic silkworms have several important properties, making them good candidates for such applications. Silk glands represent a highly efficient system for the production of large quantities of proteins, with a capacity of more than 500 mg of silk protein/larva. Also, the larval fat body is able to synthesize about 100 mg of hemolymph protein/larva. Other advantages include the low cost of silkworm rearing (less than 5 cents per larva) and the short time required for the generation of transgenic silkworms (60 days). Transgenic silkworms also allow the development of different protein production systems for various purposes (Tomita 2011;Tatemastu et al. 2012).

The current recombinant protein production systems using transgenic silkworms utilize mainly silk glands. The silk gland secretory products are categorized into two groups—sericins and fibroins. The sericins are glue proteins coating the surface of the silk thread and contribute as much as 25% of the cocoon silk. The fibroins form the silk thread and constitute the remaining 75% of the cocoon silk proteins. The sericins are produced in the middle silk gland (MSG) region, while the fibroins are secreted in the posterior part of the silk glands (PSG). The sericins and fibroins have different properties; the sericins are easily dissolved in ordinary buffer, but dissolving fibroins requires the use of strong protein-denaturing agents. The protein secretory mechanisms also differ in the MSG and PSG. The recombinant proteins produced in the MSG are secreted more easily compared to those in the PSG. For example, intact human collagen produced in the PSG is not transferred to the lumen, whereas there is no problem with its secretion in the MSG (Adachi et al. 2010;Tomita 2011). Therefore, the MSG as a production system is more useful for proteins, which require easy purification without losing their biological activity, whereas the PSG seems to be more suitable for the production of specialized proteins, like modified silks (Tatemastu et al. 2012). Consistently, human serum albumin, mouse IgG antibody, and full-length collagen have been produced in MSGs (Ogawa et al. 2007;Iizuka et al. 2009;Adachi et al. 2010). Projects involving the production of fluorescent color proteins, spider silks, cytokine, human growth factor and mini-collagen as a fusion protein with fibroin H or L chains were performed in PSGs (Iizuka et al. 2013;Teule et al. 2012;Kurihara et al. 2007;Tomita et al. 2003). The production of human μ-opioid receptor was also reported in the fat body (Tateno et al. 2009). Although the production of recombinant proteins in transgenic silkworms has already been established, problems regarding increasing productivity and posttranslational modifications need to be addressed (Tatemastu et al. 2012).

The sequence context motif surrounding the ATG initiation codon (ATG-context) is an important factor that increases protein production (Kozak [Bibr CR17][Bibr CR18];Cavener 1987;Cavener and Ray 1991;Ranjan and Hasnain 1995;Sugio et al. 2010;Agarwal et al. 2009;Cherbas and Cherbas 1993). However, there is no study on the effects of this sequence in the recombinant protein production systems of the transgenic silkworm. The optimal ATG-context motifs that show the highest efficiency in mRNA translation differ among protozoa, yeasts, vertebrates, invertebrates, and plants (Seeber 1997;Kozak 1984,1991;Joshi et al. 1997;Cavener and Ray 1991;Mankad et al. 1998). In vertebrates, the 13-base nucleotide sequence surrounding the ATG is important, and has been determined to be the GCCGCC(A/G)CCatgG motif, also called the Kozak sequence (Kozak [Bibr CR17][Bibr CR18]). Positions -3 and +4 (the A of the ATG initiation codon is marked as +1) are the most important nucleotide positions of the Kozak sequence; mutation of the nucleotides at these positions significantly reduces the efficiency of translation (Kozak 1986). The optimal sequence context in insects is completely different from that in vertebrates. In *Drosophila melanogaster*, the consensus sequence surrounding ATG is (C/A)AA(A/C)atg (Cavener 1987) and reports of the function of the context sequence are rare, with the exception of one report regarding the investigation of the effects of point mutations in the ATG-context motif of the *Drosophila* alcohol dehydrogenase gene (Feng et al. 1991). In that report, it was shown that the mutant containing the A to T substitution at position -3 showed a 2.4-fold reduction in translation efficiency, whereas five other mutations in the ATG-context motif showed 5.9- to 12.5-fold reductions compared to the original ATG-context. In Lepidoptera, sequence contexts were examined only in baculoviral protein expression systems. It was reported that the consensus ATG-context motifs in *Spodoptera frugiperda* and *B. mori* are (A/G)NC(C/T)N(A/C)CA(A/C)(C/G)atg(G/A) and ANCAAAatg, respectively (Sano et al. 2002;Chang et al. 1999).

In the present study, we compared the 14-base sequence context motif at positions -10 to +4 of the ATG initiation codon of 50 *B. mori* genes and determined the consensus as well as the least-common motif. The observed ATG-context motifs differed from those of vertebrates, but were similar to those of *D. melanogaster* and Lepidoptera described earlier (Cavener 1987;Chang et al. 1999;Sano et al. 2002). To evaluate their effects, we constructed a series of expression vectors containing luciferase or EGFP reporter genes together with various ATG-context motifs, including the novel *B. mori* consensus, variations thereof, a sericin 1 gene ATG-context, and the vertebrate Kozak sequence. Finally, we examined the effects of these ATG-context motifs on the production of the recombinant proteins in cultured cells and in the transgenic silkworms.

## Results

### Consensus sequence surrounding the ATG initiation codon in 50 B. mori genes

**Figure 1:**
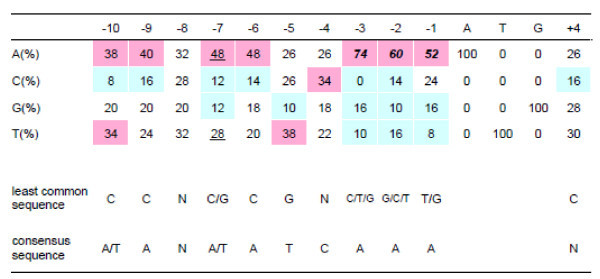
**Compilation of the 14-base sequence context of the ATG initiation codon of** ***B. mori*** **genes.** Sequences surrounding the ATG initiation codon were compiled from 50 genes in GenBank. For reference, the ATG initiation codon corresponds to +1 through +3. The frequency of each nucleotide at each position is presented as a percentage. Solo consensus (italicized letter) and co-consensus nucleotides (underlined letter) were determined by the 50/75 rule (Cavener 1987). The consensus and least-common nucleotides determined by a binominal test are indicated by red and blue boxes, respectively. The consensus and least-common nucleotides were determined using Cavener’s 50/75 rule and/or by a binominal test and are shown at the bottom. The accession numbers of genes used for the analysis are as follows: AY769299, BMU06073, D10953, D90454, DQ311154, DQ311189, DQ311242, DQ311250, DQ311264, DQ311306, DQ311321, DQ311322, DQ311328, DQ311332, DQ311333, DQ311340, DQ311341, DQ311350, DQ311356, DQ311358, DQ311360, DQ311363, DQ311365, DQ311378, DQ311384, DQ311386, DQ311388, DQ311397, DQ311402, DQ311407, DQ311412, DQ311418, DQ311430, DQ311436, DQ311438, DQ311439, DQ343760, DQ358079, DQ424947, M64336, NM_001044023, NM_001044041, NM_001113262, NM_001145941, S74376, S77508, U30289, U94993, X74320, and X95604.

To determine the consensus ATG-context motif we compared the 14-base initiation codon context motifs of genes encoding fibroin H chain, fibroin L chain, fibrohexamerin, sericin 1, and 46 other randomly selected *B. mori* genes. The consensus motif obtained from the alignment showed that ‘A’ was the most frequent nucleotide at positions -10, -9, -7, -6, -3, -2, and -1 (A of the ATG initiation codon corresponds to +1) within the context sequences of -10 to -1 using the binominal test (Figure 1). Specifically, the frequencies of ‘A’ at positions -3, -2 and -1 were higher than 50% and each was identified as a solo consensus nucleotide position by analysis of the 50/75 rule (Cavener 1987), while nucleotide ‘A’ at -7 was considered a co-consensus. From the analysis, the consensus motif (PC) was determined as (A/T)AN(A/T)ATCAAAatgN and the least-common motif, serving as a negative control (NC), was CCN(C/G)CGN(C/T/G)(G/C/T)(T/G)atgC.

### Effects of ATG initiation codon sequence context on reporter genes in BmN4 cells

**Figure 2:**
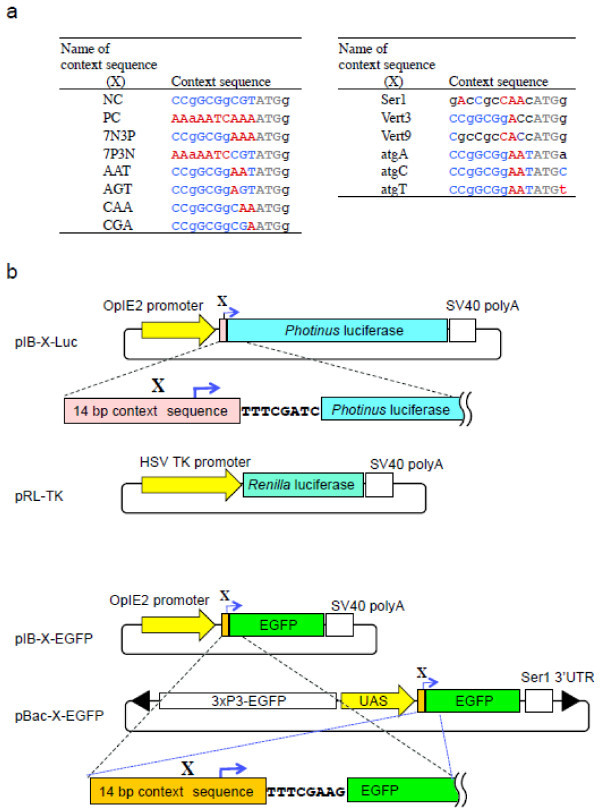
**Construction of expression vectors. a**. 14-base context motifs used in the experiment. Red and blue letters indicate the most- and least-frequent nucleotides at each position, respectively. Capital letters indicate the nucleotide of the consensus or the least-common nucleotide at each position. **b**. Physical maps of plasmids including a sequence around the translation initiation site of luciferase and EGFP constructs. X represents the newly designed context motif and the arrow points to translation initiation.

To examine the effects of the ATG-context motif, we designed a number of variants of context sequences (shown in Figure 2a). The context motifs PC and NC were selected to represent the consensus *B. mori* context and the least-common motif, respectively. 7N3P and 7P3N were designed to compare the effects of nucleotides from -1 to -3 and -4 to -10 between the least-common and consensus motifs, respectively. AAT, AGT, CAA, and CGA were designed to identify the most important nucleotide at positions -1 to -3. Ser1 is the context sequence from the sericin 1 gene. Vert3 and Vert9 were generated to determine the effects of vertebrate Kozak sequences at positions -1 to -3 and -1 to -9. The context sequences of atgA, atgC, and atgT were designed to examine the effects of the nucleotides at position +4.

We constructed vectors containing the *Photinus* luciferase gene with various context motifs (Figure 2b). When the *Photinus* luciferase vector was transfected together with the *Renilla* luciferase vector (internal control) into BmN4 cells and the activity was measured (Table 1), the *Renilla* luciferase activity appeared as constant values in each experiment, whereas the *Photinus* luciferase activity varied in the experiments, indicating that the transfection efficiency was similar in each experiment and that the difference in *Photinus* luciferase activity was caused by the variations in the context motifs. When we compared the normalized activity of the constructs (by the relative value of *Photinus* luciferase activity/*Renill*a luciferase activity), the highest activity was found in the PC-Luc construct, while NC-Luc had the lowest activity (Table 1). The activity of PC-Luc was approximately 10-fold higher than that of NC-Luc, indicating that the context motif is a critical factor affecting the activity of *Photinus* luciferase in BmN4 cells. 7N3P-Luc and 7P3N-Luc constructs demonstrated similar activities, which were ~65% of the PC-Luc activities, suggesting that the sequences from position -1 to -3 and -4 to -10 have similar effects, and that both sequences are important for luciferase expression. To examine the effects of ‘A’ at positions -1, -2, and -3 of the sequence context, five context motifs (7N3P, AAT, AGT, CAA, and CGA) were compared. Mutations of nucleotide ‘A’ at any of these positions reduced the activity, and a prominent reduction occurred when ‘A’ at position -3 was mutated, indicating that this position was the most important for expression. The effect of the nucleotide at position +4 was examined by comparing the effects among four sequence contexts (AAT, atgA, atgC, and atgT), but no significant differences were found among the four. The two sequence contexts derived from the Kozak sequence and the Ser1 context motif showed intermediate levels of activity, indicating that in BmN4 cells their effects on expression are weaker than that of the *B. mori* consensus motif.

**Table 1:** **Effects of the ATG initiation codon sequence context of** ***Photinus*** **luciferase constructs on** ***Photinus*** **luciferase expression in BmN4 cells**

Context name	***Photinus*** luciferase activity (×10 ^4^ ) (A)	***Renilla*** luciferase activity (×10 ^2^ ) (B)	***Photinus*** / ***Renilla*** (A/B)	Fold activity (%)
NC-Luc	8.3 ± 1.1	4.26 ± 0.7	196.5 ± 15.0	100
PC-Luc	88.3 ± 26.6	4.34 ± 0.5	2,008.7 ± 393.1	1,022
7N3P-Luc	55.9 ± 20.4	4.23 ± 0.5	1,307.5 ± 393.6	665
7P3N-Luc	56.8 ± 10.2	4.49 ± 0.8	1,272.5 ± 184.9	648
AAT-Luc	38.7 ± 27.4	4.44 ± 0.6	834.6 ± 539.9	425
AGT-Luc	39.4 ± 6.7	4.28 ± 0.6	941.6 ± 252.9	479
CAA-Luc	15.0 ± 2.7	4.80 ± 0.4	316.8 ± 84.3	161
CGA-Luc	11.1 ± 2.4	4.82 ± 0.4	228.2 ± 30.8	116
Ser1-Luc	45.0 ± 24.1	4.68 ± 0.6	928.2 ± 373.2	472
Vert3-Luc	25.5 ± 3.1	4.66 ± 0.3	547.9 ± 61.0	279
Vert9-Luc	36.9 ± 6.6	4.81 ± 0.3	775.4 ± 173.8	395
atgA-Luc	24.3 ± 16.9	4.47 ± 0.5	550.6 ± 413.8	280
atgC-Luc	34.3 ± 2.1	4.56 ± 0.5	759.1 ± 102.1	386
atgT-Luc	27.9 ± 12.5	4.58 ± 0.8	594.0 ± 171.2	302

Values represent the mean ± standard deviation (SD) obtained from three individual experiments.

The fold efficiency is indicated as the activity of NC equal to 100. Measurement of each luciferase activity was performed at least three times. The value of *Photinus* luciferase activity/*Renilla* luciferase activity was calculated in each measurement.

To investigate the effect of the context motif on translational efficiency, we transfected EGFP constructs bearing 11 different sequence contexts (Figure 2a; Table 2) into BmN4 cells. Transfected cells were divided into two aliquots: one to measure the amount of EGFP mRNA and the other to measure the amount of EGFP protein (Table 2). In the experiment, the amounts of EGFP mRNA and ribosomal protein 49 (rp49) mRNA (internal control) were not significantly different. This indicates that the efficiency of transcription in each construct was constant, and that the difference in the amount of EGFP protein was caused by the translational efficiency of the sequence context of EGFP mRNA. When we compared the translational efficiency of various constructs, the highest amount of EGFP protein was observed in the PC-EGFP transgene, which was ~4-fold higher than that of NC-EGFP. This indicates that the sequence context of the ATG initiation codon significantly affects translational efficiency. The translational efficiencies of 7N3P-EGFP and 7P3N-EGFP showed intermediate values between PC-EGFP and NC-EGFP. In a comparison of 7N3P-EGFP, AAT-EGFP, AGT-EGFP, CAA-EGFP, and CGA-EGFP constructs, the CGA construct demonstrated lower activity compared to the other four, indicating that the mutation of ‘A’ at positions -3 and -2 resulted in a significant reduction in translational efficiency. The Ser1-EGFP, Ver3-EGFP, and Ver9-EGFP constructs showed intermediate efficiencies, indicating that these context motifs were suboptimal for the initiation of translation in BmN4 cells. These results were similar to those obtained with the *Photinus* luciferase construct; the optimal context for luciferase and EGFP construct expression in BmN4 cells was the *B. mori* consensus motif.

**Table 2:** **Effects of the ATG initiation codon sequence context of EGFP constructs on translational efficiency of EGFP in cultured cells**

Context name	EGFP protein (μg/well) (A)	EGFP mRNA (×10 ^−6^ ) (pmol/ng) (B)	rp49 mRNA (×10 ^−7^ ) (pmol/ng) (C)	EGFP/rp49 mRNA (B/C)	Translational efficiency (A/{B/C})	Fold efficiency (%)
NC-EGFP	1.60 ± 0.5	4.31 ± 1.2	6.99 ± 0.17	6.15 ± 1.5	0.28 ± 0.13	100
PC-EGFP	6.37 ± 1.7	3.97 ± 1.5	6.68 ± 0.57	5.92 ± 1.9	1.20 ± 0.60	424
7N3P-EGFP	4.15 ± 0.6	3.47 ± 1.3	7.00 ± 0.17	4.92 ± 1.7	0.94 ± 0.41	341
7P3N-EGFP	3.54 ± 0.7	3.50 ± 1.1	6.81 ± 0.23	5.14 ± 1.6	0.76 ± 0.34	274
AAT-EGFP	4.58 ± 1.2	4.32 ± 1.2	6.89 ± 0.47	6.22 ± 1.5	0.79 ± 0.34	286
AGT-EGFP	2.72 ± 0.7	3.43 ± 1.3	6.82 ± 0.25	5.00 ± 1.7	0.61 ± 0.29	216
CAA-EGFP	4.04 ± 1.0	4.26 ± 1.2	6.63 ± 0.14	6.43 ± 1.9	0.68 ± 0.30	248
CGA-EGFP	1.20 ± 0.2	3.21 ± 1.4	6.93 ± 0.50	4.60 ± 1.8	0.30 ± 0.14	106
Ser1-EGFP	3.17 ± 0.7	3.40 ± 1.0	6.74 ± 0.31	5.03 ± 1.4	0.68 ± 0.28	248
Vert3-EGFP	2.87 ± 0.7	3.67 ± 1.2	7.20 ± 0.27	5.09 ± 1.6	0.62 ± 0.26	224
Vert9-EGFP	2.84 ± 0.3	3.30 ± 1.5	7.02 ± 0.49	4.65 ± 1.8	0.68 ± 0.27	251

Values represent the mean ± SD obtained from more than three individual experiments. The fold efficiency is indicated as the activity of NC equal to 100. Measurements of the amount of protein and mRNA were performed at least three times. Values of relative EGFP mRNA and translational efficiency were calculated in each measurement.

### Effects of ATG-context motifs on the efficiency of mRNA translation in MSG, PSG, and fat body of transgenic silkworm

**Figure 3:**
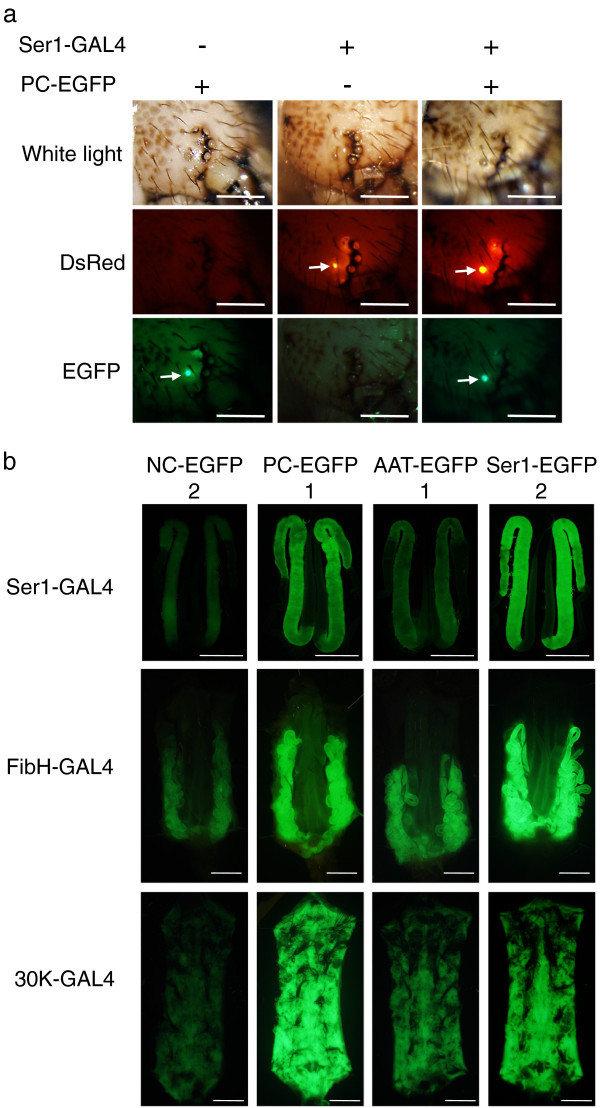
**Expression of EGFP in the transgenic silkworm. a**. Stemmata of the fifth instar larva. UAS-EGFP constructs with NC, PC, AAT, and Ser1 sequence contexts of the initiation codon were marked with a 3 × P3-EGFP marker construct allowing the expression of EGFP in the stemmata. The GAL4 driver construct under the control of the sericin 1, fibroin H chain, or 30 k protein gene promoter was linked with a marker construct, 3 × P3-DsRed, allowing the expression of DsRed in the stemmata. The stemmata of a larva harboring PC-EGFP, Ser1-GAL4, or both PC-EGFP and Ser1-GAL4 are shown. The images were taken under white light or under a fluorescence microscope equipped with an EGFP or DsRed filter. Arrows indicate the stemmata with EGFP or DsRed fluorescence. Scale bar: 1 mm. **b**. EGFP expression in the MSG, PSG, and the fat body of the transgenic silkworm with different GAL4 driver constructs. MSG, PSG, and the fat body on the sixth day of the fifth instar are shown. Images were taken under a fluorescence microscope equipped with an EGFP filter. Expression of UAS-EGFP with different context motifs was only observed in silkworms with GAL4 drivers. The numbers above the tissue photographs indicate the transgenic silkworm lines. Scale bar: 10 mm.

To examine whether the results obtained in BmN4 cells can be applied to the production of recombinant protein in the transgenic silkworm, we generated transgenic silkworms containing the EGFP constructs with the NC, PC, AAT, and Ser1 context motifs using the *piggyBac* vector pBac-X-EGFP, as shown in Figure 2b. We chose the NC, PC, and AAT context motifs because they showed the lowest, highest, and intermediate efficiencies in BmN4 cells, respectively. We also examined the Ser1 context motif originating from the sericin 1 gene, because we expected it to be adapted for the MSG translation. We generated three lines of transgenic silkworms for NC-EGFP and two lines each for PC-EGFP, AAT-EGFP, and Ser1-EGFP by the ordinary methods using the transposon *piggyBac* as a vector. Because the reporter gene is under the control of the UAS sequence (Figure 2b), the EGFP gene is not expressed without the presence of GAL4 protein. To express these four constructs in different tissues, each transgenic strain was mated with the Ser1-GAL4 (Tatematsu et al. 2010), FibH-GAL4 (Sezutsu et al. 2009), or 30 k-GAL4 lines (H. Sezutsu, personal communication), which express the GAL4 gene at the fifth instar in the MSG, PSG, and the fat body, respectively. Silkworms that possessed both the UAS-X-EGFP (Figure 2b) and GAL4 constructs were easily selected by observing the larval stemmata because the UAS and GAL4 constructs were marked with 3 × P3-EGFP and 3 × P3-DsRed, respectively (Figure 3a). When comparing the effects of the four context sequences on EGFP expression, the transgenic strains with the NC-EGFP/Ser1-GAL4, PC-EGFP/Ser1-GAL4, and AAT-EGFP/Ser1-GAL4 constructs showed weak, strong, and intermediate levels of EGFP fluorescence in MSG, respectively (Figure 3b). The strongest fluorescence was observed in the Ser1-EGFP/Ser1-GAL4 silkworm in the MSG. Similar results were observed in PSG (Figure 3b); regarding the level of expression among the four constructs, the highest was Ser1-EGFP/FibH-GAL4, the lowest was NC-EGFP/FibH-GAL4, and the PC-and AAT-EGFP constructs had intermediate expression levels in the PSG. However, the effect of the sequence context on expression in the fat body was different (Figure 3b); the PC construct showed higher activity than that of Ser1-EGFP, and the expression levels of the NC and AAT constructs were much lower than those of PC and Ser1.

To further analyze the effects of the context sequence, we measured the amounts of EGFP protein and mRNA in the MSG (Table 3), PSG (Table 4), and fat body (Table 5), and compared the translational efficiency among the four constructs. In the MSG, the highest translational efficiency was observed for the Ser1-EGFP construct and the lowest in NC-EGFP. PC-EGFP also showed very high translational efficiency, while AAT-EGFP was intermediate. The translational efficiency of Ser1-EGFP was ~20-fold higher than NC-EGFP, suggesting that the context motif of the ATG initiation codon is a critical factor for increasing the yield of recombinant proteins in transgenic silkworms. An amount of the EGFP protein proportional to the translational efficiency was produced from each construct in the MSG. The rank order of protein amounts was Ser1-, PC-, AAT- and NC-EGFP. Similar results were observed in the PSG with the same rank order of individual constructs: Ser1 > PC > AAT > NC-EGFP. The increase in the translational efficiency of Ser1-EGFP was 6-fold in the PSG when compared to the lowest-activity construct, NC-EGFP. In addition, the translational efficiency of each construct in the PSG was much higher than that in the MSG, and the protein yield was almost proportional to the translational efficiency. In the fat body, the rank order of the translational efficiency was PC > Ser1 > AAT > NC-EGFP. PC-EGFP was almost 50-fold higher in the translational efficiency compared to NC-EGFP. The EGFP protein yield was proportional to this order, suggesting that the context sequence was critical for recombinant protein production. NC-EGFP demonstrated the lowest EGFP protein production and translational efficiency in all tissues examined. Although PC-EGFP showed the highest EGFP protein production and translational efficiency in the fat body, the construct with the highest efficiency in the MSG and PSG was Ser1-EGFP. Thus, the effects of each sequence context on EGFP expression in the fat body were similar to those observed in BmN4 cells, but differed from the effects in MSG and PSG.

**Table 3:** **Effects of sequence context of the ATG initiation codon on translational efficiency of EGFP expressed in MSG**

	Line no.	EGFP protein (μg/larva) (A)	EGFP mRNA (×10 ^−7^ ) (pmol/ng) (B)	rp49 mRNA (×10 ^−7^ ) (pmol/ng) (C)	EGFP/rp49 mRNA (B/C)	Translational efficiency (A/{B/C})	Fold efficiency (%)
NC	1	31.4 ± 5.8	70.8	7.83	9.05	3.47	
-EGFP	2	16.8 ± 2.4	44.1	7.26	6.07	2.76	
	3	12.6 ± 1.7	49.9	7.70	6.48	1.95	
	Average	20.3	54.9	7.60	7.23	2.73	100
PC	1	130.2 ± 9.9	19.8	6.96	2.85	45.7	
-EGFP	2	158.9 ± 12.1	29.6	6.71	4.40	36.1	
	Average	144.5	24.7	6.84	3.61	40.9	1,498
AAT	1	76.8 ± 7.2	48.7	9.04	5.39	14.2	
-EGFP	2	89.0 ± 12.9	34.4	8.14	4.23	21.0	
	Average	82.9	41.6	8.59	4.84	17.6	645
Ser1	1	276.3 ± 36.4	40.8	8.14	5.01	55.1	
-EGFP	2	261.4 ± 18.4	37.3	7.76	4.80	54.4	
	Average	268.9	39.0	7.95	4.91	54.8	2,007

Values obtained from each line and averages of lines are shown. The fold efficiency is indicated as the efficiency of the average of NC equal to 100.

**Table 4:** **Effects of sequence context of the ATG initiation codon on translational efficiency of EGFP expressed in PSG**

	Line no.	EGFP protein (μg/larva) (A)	EGFP mRNA (×10 ^−7^ ) (pmol/ng) (B)	rp49 mRNA (×10 ^−7^ ) (pmol/ng) (C)	EGFP/rp49 mRNA (B/C)	Translational efficiency (A/{B/C})	Fold efficiency (%)
NC	1	12.9 ± 0.8	4.20	3.95	1.06	12.1	
-EGFP	2	9.9 ± 0.3	1.73	3.54	0.49	20.2	
	3	12.6 ± 0.7	0.48	3.60	0.14	93.0	
	Average	11.8	2.14	3.70	0.56	41.8	100
PC	1	80.6 ± 15.3	1.35	2.93	0.46	175.5	
-EGFP	2	104.8 ± 24.2	2.14	3.65	0.59	178.1	
	Average	92.7	1.75	3.29	0.52	176.8	423
AAT	1	25.5 ± 3.6	1.64	3.23	0.51	50.3	
-EGFP-EGFP	2	53.5 ± 15.8	0.84	3.50	0.24	220.5	
	Average	39.5	1.24	3.37	0.37	135.4	324
Ser1	1	94.4 ± 10.4	1.53	3.52	0.44	216.9	
-EGFP	2	106.2 ± 34.7	1.15	3.09	0.37	284.9	
	Average	100.3	1.34	3.31	0.40	250.9	600

Values obtained from each line and averages of lines are shown. The fold efficiency is indicated as the efficiency of the average of NC equal to 100.

**Table 5:** **Effects of sequence context of the ATG initiation codon on translational efficiency of EGFP expressed in the fat body**

	Line no.	EGFP protein (μg/larva) (A)	EGFP mRNA (×10 ^−7^ ) (pmol/ng) (B)	rp49 mRNA (×10 ^−7^ ) (pmol/ng) (C)	EGFP/rp49 mRNA (B/C)	Translational efficiency (A/{B/C})	Fold efficiency (%)
NC	1	4.9 ± 0.6	25.8	5.15	5.01	0.98	
-EGFP	2	4.4 ± 0.1	13.7	3.14	4.37	1.01	
	3	1.3 ± 0.1	21.6	3.86	5.61	0.23	
	Average	3.5	20.4	4.05	5.04	0.70	100
PC	1	51.8 ± 3.0	11.5	6.13	1.87	27.6	
-EGFP	2	70.2 ± 17.3	12.4	6.89	1.80	39.0	
	Average	61.0	11.9	6.51	1.83	33.3	4,757
AAT	1	11.9 ± 0.1	15.6	4.20	3.72	3.20	
-EGFP	2	4.2 ± 0.2	11.8	4.83	2.44	1.71	
	Average	8.0	13.7	4.52	3.03	2.65	379
Ser1	1	44.6 ± 3.8	35.3	6.20	5.70	7.82	
-EGFP	2	50.6 ± 2.5	11.5	6.08	1.90	26.7	
	Average	47.6	23.4	6.14	3.82	12.5	1,785

Values obtained from each line and averages of lines are shown. The fold efficiency is indicated as the efficiency of the average of NC equal to 100.

## Discussion

We performed a compilation of the 14-nucleotide sequence motifs surrounding the ATG initiation codon of 50 *B. mori* genes and determined the consensus ATG context motif (A/T)AN(A/T)ATCAAAatgN, as well as the least-common ATG context CCN(C/G)CGN(C/T/G)(G/C/T)(T/G)atgC, which served as the control. The novel consensus motif was consistent with the previously reported consensus motifs of *B. mori* (Chang et al. 1999 (Cavener ) and *Drosophila*^1987^), but differed from that of vertebrates (Kozak [Bibr CR17][Bibr CR18]). When we compared the previously reported *B. mori* consensus motif (ANCAAAatgNNN) with our novel consensus sequence, a significant difference was found in the frequency of ‘T’ at position -5. The frequency of ‘T’ in our results was significantly higher than would be expected for a random occurrence (Figure 1). Our *B. mori* consensus motif was slightly more similar to the *D. melanogaster* consensus motif, (C/A)AA(A/C)atg, reported by Cavener (1987). When we reanalyzed Cavener’s original data using our method, we received the consensus AANAAN(C/A)AA(A/C)atg. Differences between our *B. mori* consensus motif and the reanalyzed *D. melanogaster* consensus motif were at positions -1, -4, -5, -7, and -10.

We also determined which nucleotides of the consensus context motifs were important for efficient translation. Our results show that ‘A’ at position -3 was the most important nucleotide; substitution of this nucleotide caused a significant reduction in efficiency. The region of -10 to -4 also displayed a significant effect, while the nucleotide at position +4 did not have any detectable effect. Our results differ from those of Chang et al. (1999), who found that the nucleotides at positions -1 to -6 had no effect while those at +4 to +6 significantly increased the efficiency. Furthermore, we found that our new consensus motif of *B. mori* genes was more efficient than the Kozak sequence. However, Sano et al. (2002) reported that the use of the 5′-untranslated region including the Kozak sequence dramatically increased expression levels in baculovirus-infected cells. In both of these studies, baculovirus was used for transgene expression, whereas we used transient expression of plasmid. The differences between our results and previously reported data may be due to the different expression systems used in experiments; further studies are required to determine the exact reasons for the differences.

Three features differed between the vertebrate consensus Kozak motif and our *B. mori* consensus motif. First, the vertebrate ATG context shows a strong preference for nucleotide position +4 (Kozak [Bibr CR17][Bibr CR18][Bibr CR20]), but not in *B. mori*. Second, the vertebrate consensus motif is CG-rich throughout the sequence, but A-rich in *B. mori*. Third, three upstream triplet repeats, RCCRCCRCCatg, which are important for ribosomal recognition, are present in vertebrates (Kozak 1987b), while no such repeats were found in our results, suggesting that mRNA recognition by the ribosome differs between insects and vertebrates.

The assays of context motif effects demonstrated that these sequences significantly affect the efficiency of translation initiation in BmN4 cultured cells, as well as the production of recombinant proteins in the MSG, PSG, and fat body of transgenic silkworms. This is the first report that optimization of context motifs is important for efficient recombinant protein production in transgenic silkworms. Such optimization is simple and does not affect the protein sequence. In addition, context motifs can be used for the suppression of translation initiation when a reduction in protein production is required. Furthermore, context motifs may facilitate precise translation initiation. Kanamori et al. (2010) reported that a leaky scanning mechanism for translation initiation sometimes causes the utilization of an internal ATG codon in *B. mori* cells.

In our experiment, the influence of context motifs on translational efficiency varied depending on the tissue. We designed NC and PC sequence contexts that represented the least-common and consensus sequences, respectively. The translational efficiency of the NC sequences was the lowest in all tissues, whereas the PC sequences demonstrated the highest efficiency in BmN4 cells and the fat body, but not in the MSG and PSG. The BmN4 cell line is derived from silkworm ovaries; however, it may retain similar characters to fat body cells because dexamethasone treatment induces accumulation of lipid in the cells (Akiduki and Imanishi 2007). Feng et al. (1991) reported that the effects of context sequences on translation in *D. melanogaster* were stage-dependent. These results suggest that stage- and tissue-specific adjustment of context motifs is needed for the maximum production of recombinant proteins. Silk glands represent an organ that is highly specialized for massive production of several secretory proteins within a short time interval (Julien et al. 2005). Our data show that this specialization also involves the adjustment of ATG initiation codon context motifs.

The optimization of different tissue-specific expression systems in transgenic silkworms is important from the viewpoint of posttranslational modifications, including glycosylations. The recombinant proteins produced by insect cells generally carry paucimannose-type n-glycans (Harrison and Jarvis 2006). The proteins produced by tissues other than the silk gland also have paucimanmose- or highmannose-type n-glycans (Tomita 2011). However, the recombinant proteins produced in the MSG contain N-acetylglucosaminylated complex N-glycans (Iizuka et al. 2009). The optimization of the ATG context motifs is part of a large project developing recombinant protein production systems adapted for different tissues.

In conclusion, we identified the ATG context motif consensus in the silkworm *B. mori*, and showed that optimization of the sequence context is useful for increased production of recombinant protein in cultured cells and in transgenic silkworms. The context motifs best adapted to high production in cultured cells and the fat body differed from those in the MSG and PSG. Therefore, different optimized context sequences for different tissues may be required for the maximum expression of transgenes.

## Methods

### Construction of expression vectors

To generate the plasmid pIB-X-EGFP (Figure 2b), the EGFP gene amplified from the plasmid pBac[UAS-ser_sig-EGFP/3 × P3-EGFP] (Tatematsu et al. 2010) using the primers EGFP-BstBI-U and EGFP Stop(+)L (Table 6) was inserted into the *Bam*HI-*Eco*RI site of pBluescript SK(-) (TOYOBO, Osaka, Japan). The constructed plasmid, pEGFP/pBS, contained the T*TTCGAA*G (italicized letter, BstBI site) sequence downstream of the context sequence instead of the original EGFP sequence TGAGCAAG (this sequence change did not alter the amino acid sequence of EGFP). The adapter, except for IB-BlnI in Table 6, was inserted into the *Bst*BI-*Bam*HI site of the plasmid pEGFP/pBS to generate the plasmid pBS-X-EGFP. Simultaneously, the IB-BlnI adapter (Table 6) was inserted into the *Hind*III-*Kpn*I site of the plasmid pIB-V5/His (Life Technologies, Carlsbad, CA, USA) to introduce a *Bln*I site, and the resultant plasmid pIB-V5/His_BlnI was obtained. Then, the NheI fragment from pBS-X-EGFP was inserted into the *Bln*I site of the pIB-V5/His_BlnI. To generate the plasmid pBac-X-EGFP (Figure 2b), the NheI fragment from the plasmid pBS-X-EGFP was inserted into the *Bln*I site of the plasmid pBac[SerUAS/3 × P3-EGFP] (Tatematsu et al. 2010).

**Table 6:** **List of primers and adapters used in the experiments**

Name	Sequences
Primer	
EGFP-BstI-U	5′-GGGATCCGCTAGCACCATGGTTTCGAAGGGCGAG-3′
EGFP-Stop(+)-L	5′-GGAATTCGGACCGCTAGCTTACTTGTACAGCTCG-3′
pGL3-U	5′-GGATCGATCAAAAACATAAAGAAAGGCCCGGCG-3′
pGL3-L	5′-GGGCTAGCTTACACGGCGATCTTTCCGCCCTTC-3′
EGFPLCU2	5′-AACTTCAAGATCCGCCACAACATCGAGGAC-3′
EGFPLCL2	5′-AGGACCATGTGATCGCGCTTCTCGT-3′
rp49LCF2	5′-GGATCGCTATGACAAACTTAAGAGGA-3′
rp49LCR1	5′-TGCTGGGCTCTTTCCACGA-3′
Adapter	
NC	5′-GATCCGCTAGCCCGGCGGCGTATGGTTT-3′
	3′-GCGATCGGGCCGCCGCATACCAAAGC-5′
PC	5′-GATCCGCTAGCAAAAATCAAAATGGTTT-3′
	3′-GCGATCGTTTTTAGTTTTACCAAAGC-5′
7N3P	5′-GATCCGCTAGCCCGGCGGAAAATGGTTT-3′
	3′-GCGATCGGGCCGCCTTTTACCAAAGC-5′
7P3N	5′-GATCCGCTAGCAAAAATCCGTATGGTTT-3′
	3′-GCGATCGTTTTTAGGCATACCAAAGC-5′
AAT	5′-GATCCGCTAGCCCGGCGGAATATGGTTT-3′
	3′-GCGATCGGGCCGCCTTATACCAAAGC-5′
AGT	5′-GATCCGCTAGCCCGGCGGAGTATGGTTT-3′
	3′-GCGATCGGGCCGCCTCATACCAAAGC-5′
CAA	5′-GATCCGCTAGCCCGGCGGCAAATGGTTT-3′
	3′-GCGATCGGGCCGCCGTTTACCAAAGC-5′
CGA	5′-GATCCGCTAGCCCGGCGGCGAATGGTTT-3′
	3′-GCGATCGGGCCGCCGCTTACCAAAGC-5′
Ser1	5′-GATCCGCTAGCGACCGCCAACATGGTTT-3′
	3′-GCGATCGCTGGCGGTTGTACCAAAGC-5′
Vert3	5′-GATCCGCTAGCCCGGCGGACCATGGTTT-3′
	3′-GCGATCGGGCCGCCTGGTACCAAAGC-5′
Vert9	5′-GATCCGCTAGCCGCCGCCACCATGGTTT-3′
	3′-GCGATCGGCGGCGGTGGTACCAAAGC-5′
atgA	5′-GATCCGCTAGCCCGGCGGAATATGATTT-3′
	3′-GCGATCGGGCCGCCTTATACTAAAGC-5′
atgC	5′-GATCCGCTAGCCCGGCGGAATATGCTTT-3′
	3′-GCGATCGGGCCGCCTTATACGAAAGC-5′
atgT	5′-GATCCGCTAGCCCGGCGGAATATGTTTT-3′
	3′-GCGATCGGGCCGCCTTATACAAAAGC-5′
IB-BlnI	5′-AGCTTCCTAGGGTCGACGGTAC-3′
	3′-AGGATCCCAGCTGC-5′

To generate pIB-X-Luc constructs (Figure 2b), the *Photinus* (firefly) luciferase gene was amplified from the plasmid pGL3 (Promega, Madison, WI, USA) using primers pGL3 U and pGL3 L (Table 6). Then, the amplified fragment was inserted into the plasmid pZErO-2 (Life Technologies) in the same direction as the *lacZ* gene. A ClaI-EcoRI fragment from pLuc/pZero was inserted into the *Bst*BI-*Eco*RI site of pBS-X-EGFP to generate pBS-X-Luc constructs. The NheI fragment of pBS-X-Luc was inserted into the *Bln*I site of the plasmid pIB-V5/His, and pIB-X-Luc was constructed. Although the N-terminal amino acid sequence of the luciferase gene was altered in the process of plasmid construction, there was little or no effect on enzymatic activity.

The sequence of the constructed plasmid was verified by DNA sequencing on an ABI3100 or ABI3130 DNA sequencer with a BigDye terminator DNA sequencing kit (Life Technologies).

### Measurement of luciferase activity and EGFP expression in BmN4 cells

To measure the effect of the ATG-context motif sequence on luciferase activity, BmN4 cells were cultured in six-well plates until they were ~80% confluent. The transfection was performed with a mixture of 1-μg pIB-X-Luc vector, 0.1-μg pRL-TK vector (Promega) and 6-μL FuGENE HD reagent (Promega). Transfected cells were lysed in 200-μL 1 × passive lysis buffer (Promega) 3 days after transfection, and luciferase activity was measured using a dual luciferase assay kit (Promega). To measure EGFP protein and mRNA, cultured BmN4 cells were transfected with 1-μg pIB-X-EGFP vector and 6-μL FuGENE HD reagent (Promega). Cells were harvested 3 days after transfection and split into two aliquots to measure mRNA and protein by quantitative PCR and enzyme-linked immunosorbent assay (ELISA), respectively. Isogen (Nippon Gene, Tokyo, Japan) was used to extract total RNA from transfected BmN4 cells, and the cDNA was synthesized from isolated RNA using RevertraAce reverse transcriptase (TOYOBO). Quantitative PCR was performed as reported previously (Tatematsu et al. 2010). The primers EGFPLCU2 and EGFPLCL2 (Table 6) were used to amplify EGFP mRNA, and primers rp49LCF2 and rp49LCR1 (Table 6) were used to amplify rp49 (Table 2). To measure EGFP protein, transfected BmN4 cells were extracted with 300-μL 1 × passive lysis buffer (Promega), and an ELISA for EGFP protein was performed on Reacti-Bind anti-GFP-coated plates (Pierce, Rockford, IL, USA), as previously reported (Tatematsu et al. 2010). The measurements of luciferase activity and EGFP protein were repeated at least three times.

### Generation of transgenic silkworms and measurement of EGFP expression in MSG, PSG, and fat body

The silkworm strain w1-pnd, which is non-diapausing, with non-pigmented eggs and eyes, was used to generate transgenic silkworms. The diapausing strain w-1 was used to maintain each transgenic strain. These strains were maintained at the Transgenic Silkworm Research Unit, National Institute of Agrobiological Sciences. Silkworm larvae were reared on an artificial diet (Nosan, Yokohama, Japan) at 25°C.

Transgenic silkworms were generated as previously reported using transposon *piggyBac* as a vector (Tamura et al. 2007;Tatematsu et al. 2010). Transgenic silkworms harboring the EGFP construct with different sequence contexts were mated with adults from Ser1-GAL4 (Tatematsu et al. 2010), FibH-GAL4 (Sezutsu et al. 2009), or 30 K-GAL4 strains (H. Sezutsu, personal communication), and their F1 larvae, harboring both EGFP and GAL4 constructs, were used for the experiment. The MSG, PSG, and fat body were dissected from 10 larvae on the sixth day of the fifth instar. The amounts of EGFP mRNA and protein in tissue were measured using quantitative PCR and ELISA, as reported previously (Tatematsu et al. 2010).

## References

[CR1] Adachi T, Wang X, Murata T, Obara M, Akutsu H, Machida M, Umezawa A, Tomita M (2010). Production of a nontriple helical collagen alpha chain in transgenic silkworms and its evaluation as a gelatin substitute for cell culture. Biotechnol Bioeng.

[CR2] Agarwal S, Jha S, Sanyal I, Amla DV (2009). Effect of point mutations in translation initiation context on the expression of recombinant human a1-proteinase inhibitor in transgenic tomato plants. Plant Cell Rep.

[CR3] Akiduki G, Imanishi S (2007). Establishment of a lipid accumulation model in an insect cell line. Archiv Insect Biochem Physiol.

[CR4] Cavener DR (1987). Comparison of the consensus sequence flanking translational start sites in Drosophila and vertebrates. Nucleic Acids Res.

[CR5] Cavener DR, Ray SC (1991). Eukaryotic start and stop translation sites. Nucleic Acids Res.

[CR6] Chang MJ, Kuzio J, Blissard GW (1999). Modulation of translational efficiency by contextual nucleotides flanking a Baculovirus initiator AUG codon. Virology.

[CR7] Cherbas L, Cherbas P (1993). The arthropod initiator: the capsite consensus plays an important role in transcription. Insect Biochem Mol Biol.

[CR8] Feng Y, Gunter LE, Organ EL, Cavener DR (1991). Translation initiation in Drosophila melanogaster is reduced by mutations upstream of the AUG initiator codon. Mol Cell Biol.

[CR9] Harrison RL, Jarvis DL (2006). Protein N-glycosylation in the baculovirus-insect cell expression system and engineering of insect cells to produce “mammalianized” recombinant glycoproteins. Adv Virus Res.

[CR10] Iizuka M, Ogawa S, Takeuchi A, Nakakita S, Kubo Y, Miyawaki Y, Hirabayashi J, Tomita M (2009). Production of a recombinant mouse monoclonal antibody in transgenic silkworm cocoons. FEBS J.

[CR11] Iizuka T, Sezutsu H, Tatematsu K, Kobayashi I, Yonemura N, Uchino K, Nakajima K, Kojima K, Takabayashi C, Machii H, Yamada K, Kurihara H, Asakura T, Nakazawa Y, Miyawaki A, Karasawa S, Kobayashi H, Yamaguchi J, Kuwabara N, Nakamura T, Yoshii K, Tamura T (2013). Colored fluorescent silk made by transgenic silkworms. Adv Funct Mater.

[CR12] Joshi CP, Zhou H, Huang Q, Chiang VL (1997). Context sequences of translation initiation codon in plants. Plant Mol Biol.

[CR13] Julien E, Coulon-Bublex M, Garel A, Royer C, Chavancy C, Pudhomme JC, Couble P, Gilbert LI, Iatrou K, Gill SS (2005). Silk gland development and regulation of silk protein genes. Comprehensive molecular insect science.

[CR14] Kanamori Y, Hayakawa Y, Matsumoto H, Yasukochi Y, Shimura S, Nakahara Y, Kiuchi M, Kamimura M (2010). A eukaryotic (insect) tricistronic mRNA encodes three proteins selected by context-dependent scanning. J Biol Chem.

[CR15] Kozak M (1984). Point mutations close to the aug initiator codon affect the efficiency of translation of rat preproinsulin in vivo. Nature.

[CR16] Kozak M (1986). Point mutations define a sequence flanking the AUG initiator codon that modulates translation by eukaryotic ribosomes. Cell.

[CR17] Kozak M (1987). An analysis of 5′-noncoding sequences from 699 vertebrate messenger RNAs. Nucleic Acids Res.

[CR18] Kozak M (1987). At least six nucleotides preceding the AUG initiator codon enhance translation in mammalian cells. J Mol Biol.

[CR19] Kozak M (1991). Structural features in eukaryotic mRNAs that modulate the initiation of translation. J Biol Chem.

[CR20] Kozak M (1997). Recognition of AUG and alternative initiator codons is augmented by G in position +4 but is not generally affected by the nucleotides in positions +5 and +6. EMBO J.

[CR21] Kurihara H, Sezutsu H, Tamura T, Yamada K (2007). Production of an active feline interferon in the cocoon of transgenic silkworms using the fibroin H-chain expression system. Biochem Biophys Res Commun.

[CR22] Mankad RV, Gimelbrant AA, McClintock TS (1998). Consensus translational initiation sites of marine invertebrate phyla. Biol Bull.

[CR23] Ogawa S, Tomita M, Shimizu K, Yoshizato K (2007). Generation of a transgenic silkworm that secretes recombinant proteins in the sericin layer of cocoon: production of recombinant human serum albumin. J Biotechnol.

[CR24] Ranjan A, Hasnain SE (1995). Influence of codon usage and translation initiation codon context in theAcNPV-based expression system: computer analysis using homologous and heterologous genes. Virus Genes.

[CR25] Sano KI, Maeda K, Oki M, Maeda Y (2002). Enhancement of protein expression in insect cells by a lobster tropomyosin cDNA leader sequence. FEBS Lett.

[CR26] Seeber F (1997). Consensus sequence of translational initiation sites from Toxoplasma gondii genes. Parasitol Res.

[CR27] Sezutsu H, Uchino K, Kobayashi I, Tatematsu K, Iizuka T, Yonemura N, Tamura T (2009). Conservation of fibroin gene promoter function between the domesticated silkworm Bombyx mori and the wild silkmoth Antheraea yamamai. J Insect Biotechnol Sericol.

[CR28] Sugio T, Matsuura H, Matsui T, Matsunaga M (2010). Effect of the sequence context of the AUG initiation codon on the rate of translation in dicotyledonous and monocotyledonous plant cells. J Biosci Bioeng.

[CR29] Tamura T, Kuwabara N, Uchino K, Kobayashi I, Kanda T (2007). An improved DNA injection method for silkworm eggs drastically increases the efficiency of producing transgenic silkworms. J Insect Biotechnol Sericol.

[CR30] Tatemastu K, Sezutsu H, Tamura T (2012). Utilization of transgenic silkworms for recombinant protein production. J Biotechnol Biomaterial.

[CR31] Tatematsu K, Kobayashi I, Uchino K, Sezutsu H, Iizuka T, Yonemura N, Tamura T (2010). Construction of a binary transgenic gene expression system for recombinant protein production in the middle silk gland of the silkworm Bombyx mori. Transgenic Res.

[CR32] Tateno M, Toyooka M, Shikano Y, Takeda S, Kuwabara N, Sezutsu H, Tamura T (2009). Production and characterization of the recombinant human μ-opioid receptor from transgenic silkworms. J Biochem.

[CR33] Teule F, Miao YG, Sohn BH, Kim YS, Hull JJ, Fraser MJ, Lewis RV, Jarvis DL (2012). Silkworms transformed with chimeric silkworm/spider silk genes spin composite silk fibers with improved mechanical properties. PNAS.

[CR34] Tomita M (2011). Transgenic silkworms that weave recombinant proteins into silk cocoons. Biotechnol Lett.

[CR35] Tomita M, Munetsuna H, Sato T, Adachi T, Hino R, Hayashi M, Shimizu K, Nakamura N, Tamura T, Yoshizato K (2003). Transgenic silkworms produce recombinant human type III procollagen in cocoons. Nat Biotechnol.

